# The Impact of Dietary Fucosylated Oligosaccharides and Glycoproteins of Human Milk on Infant Well-Being

**DOI:** 10.3390/nu12041105

**Published:** 2020-04-16

**Authors:** Magdalena Orczyk-Pawiłowicz, Jolanta Lis-Kuberka

**Affiliations:** Department of Chemistry and Immunochemistry, Wroclaw Medical University, M. Skłodowskiej-Curie 48/50, 50-369 Wrocław, Poland

**Keywords:** dietary sugars, fucosylated oligosaccharides, fucosylated glycoproteins, HMOs, human milk, infant feeding, infant well-being, secretor/non-secretor status

## Abstract

Apart from optimal nutritional value, human milk is the feeding strategy to support the immature immunological system of developing newborns and infants. The most beneficial dietary carbohydrate components of breast milk are human milk oligosaccharides (HMOs) and glycoproteins (HMGs), involved in both specific and nonspecific immunity. Fucosylated oligosaccharides represent the largest fraction of human milk oligosaccharides, with the simplest and the most abundant being 2′-fucosyllactose (2′-FL). Fucosylated oligosaccharides, as well as glycans of glycoproteins, as beneficial dietary sugars, elicit anti-adhesive properties against fucose-dependent pathogens, and on the other hand are crucial for growth and metabolism of beneficial bacteria, and in this aspect participate in shaping a healthy microbiome. Well-documented secretor status related differences in the fucosylation profile of HMOs and HMGs may play a key but underestimated role in assessment of susceptibility to fucose-dependent pathogen infections, with a potential impact on applied clinical procedures. Nevertheless, due to genetic factors, about 20% of mothers do not provide their infants with beneficial dietary carbohydrates such as 2′-FL and other α1,2-fucosylated oligosaccharides and glycans of glycoproteins, despite breastfeeding them. The lack of such structures may have important implications for a wide range of aspects of infant well-being and healthcare. In light of the above, some artificial mixtures used in infant nutrition are supplemented with 2′-FL to more closely approximate the unique composition of maternal milk, including dietary-derived fucosylated oligosaccharides and glycoproteins.

## 1. Introduction

The recommendation of breast milk as the best way to feed newborns and infants is associated with its unique composition. Mother’s milk, in addition to basic nutrients, such as proteins, fats, and sugars, contains bioactive molecules such as cytokines, enzymes, growth factors, hormones, glycoproteins, glycolipids, oligosaccharides and vitamins [1,2,3]. Moreover, breast milk is rich in molecules with immunomodulatory properties that have a significant impact on the maturation of the immune, digestive and nervous systems of breastfed newborns and infants and provide protection against pathogens. Above all, it is an indispensable element of specific and nonspecific immunity transferred to the newborn and infants along with the mother’s milk [4,5,6,7,8,9].

The most important beneficial glycoconjugates of breast milk include free milk oligosaccharides (HMOs, human milk oligosaccharides), glycoproteins involved in specific (S-IgA, secretory immunoglobulin A, IgM, immunoglobulin M and IgG, immunoglobulin G) and nonspecific immunity (LF, lactoferrin) and glycolipids [10,11,12,13,14]. HMOs present in breast milk are the third largest milk fraction after lactose and fat (human milk contains approximately 70, 41 and 5–20 g of lactose, fat and oligosaccharides per liter, respectively) [5,15,16,17]. HMOs are based on a lactose molecule (disaccharide made of a galactose (Gal) linked by a β1, 4-glycosidic bond to a glucose (Glc)) to which other monosaccharides, such as N-acetylglucosamine (GlcNAc), Gal, fucose (Fuc) and sialic acid (Neu5Ac), can be attached. HMO synthesis take place in the Golgi apparatus of follicular cells of the mammary gland. HMOs represent incredible structural diversity, despite the fact that in their qualitative composition only five different sugars occur [18,19,20]. Nevertheless, individual monosaccharides can be connected with each other by different types of O-glycosidic bonds, which results in the formation of other spatial structures, depending on the linkage position. Recent developments in glycomics led to the identification of over 200 different structures of HMOs, varying in size from three to 22 monosaccharide units [21,22,23].

Human milk glycoproteins (HMGs) are a large and very diverse group of proteins which are involved in a variety of biological processes [6,7,24]. However, in most cases the presence of N- and/or O-glycans attached to the protein core are crucial for their activity, namely N-glycans play an important role in regulating many intracellular and extracellular functions, while O-glycans form a crucial interface between epithelial cells and the external mucosal surfaces of the body. Additionally, glycans attached to HMGs can serve as prebiotic factors in the shaping of a favorable intestinal microbiota and act as effective decoy receptors for pathogens [4,6,7,24]. Moreover, the oligosaccharide chains attached to proteins affect the physicochemical properties of these molecules, including solubility, viscosity, charge, spatial structure, and greater resistance against proteolysis in the digestive tract [6,7,25]. Dietary glycoproteins of mother’s milk are divided depending on their presence into milk fat globule membrane (MFGM) or skim milk fractions [6,7,26,27]. The most abundant glycoproteins of the MFGM fraction are mucins, lactadherin, butyrophilin and lactoperoxidase; and for the skim milk fraction, they are casein, lactoferrin, secretory IgA and other immunoglobulins such as IgG and IgM, fibronectin (FN), α_1_-acid glycoprotein (AGP) and many others [6,7,27,28,29,30,31].

The review summarizes the available data concerning the composition of dietary fucosylated HMOs and HMGs in relation to milk maturation, gestational age, and maternal morbidities. The second part describes the impact of dietary fucosylated HMOs and HMGs for a wide range of aspects of infants’ well-being and healthcare with particular emphasis on their protective function and the use of donor milk. Moreover, strategies to improve the infants’ formula are presented.

## 2. Materials and Method

The initial search strategy was developed for PubMed and then adapted to the Scopus database. The literature was selected from last 20 years. The databases were systematically searched from December 2019 until 29 February 2020 and restricted to those published in English. Limitations have been applied to exclude conference papers, editorials, letters, commentaries, short surveys, and notes. Further studies were found using the reference lists of articles considered eligible for the review. Table 1 outlines the search strategies and key terms used.

## 3. Structures and Diversity of HMOs

The most important molecules in the processes of biological recognition, such as fucose and sialic acid, are a part of individual HMOs. They are attached by glycosidic bonds; fucose can be attached by α1,2- and/or α1,3/4 to Gal and GlcNAc, respectively, while sialic acid is attached by another type of glycosidic bonds, i.e., α2,3 and/or α2,6. However, it should be pointed out that, in contrast to other HMO-building monosaccharides, the unit of Fuc and Neu5Ac present in oligosaccharide structures can no longer be substituted with another monosaccharide [30,32,33,34,35,36].

**Figure 1:**
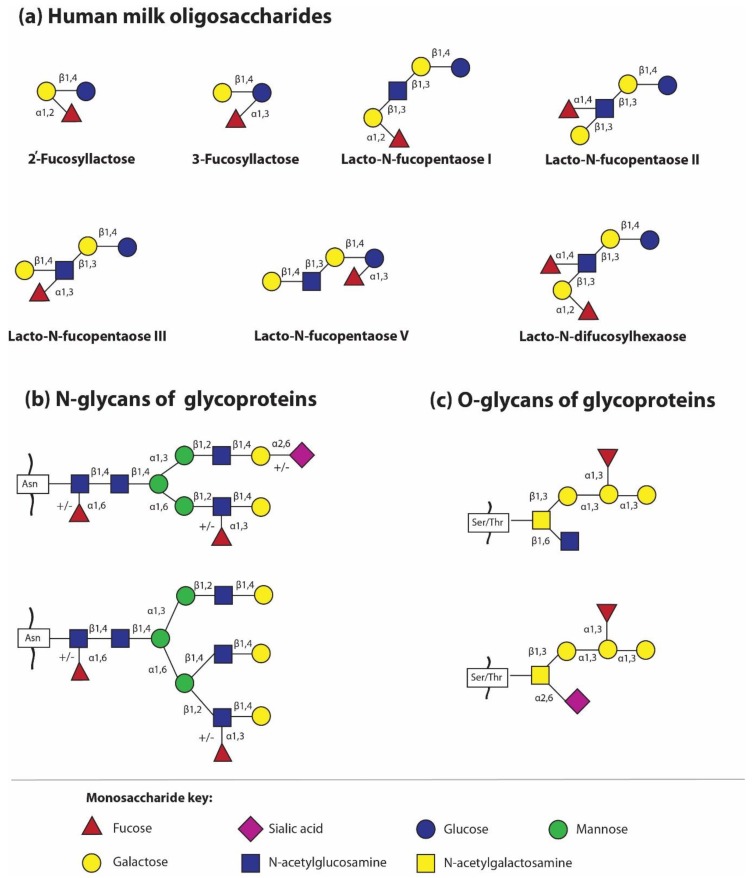
The main fucosylated structures of human milk oligosaccharides and glycans attached to glycoproteins [49,50,51]. (**a**) The most abundant fucosylated human milk oligosaccharides; (**b**) fucosylated N-glycans of glycoproteins – the two most common fucosylated structures in human milk N-glycome are presented [52,53]; Asn – asparagine; and (**c**) fucosylated O-glycans of human milk glycoproteins [52,53]; Ser – serine, Thr – threonine.

The simplest oligosaccharides present in breast milk are trisaccharides, made of lactose to which fucose or sialic acid molecules have been attached by means of various glycosidic bonds. Briefly, in this way four different oligosaccharides are formed, two after adding fucose to lactose by α1,2- fucosyltransferase and α1,3/4- fucosyltransferase, respectively, i.e., Fuc attached by an α1,2-glycosidic linkage to Gal gives 2′-fucosyllactose (2′-FL), while Fuc attached by an α1,3-glycosidic linkage gives 3-fucosyllactose (3-FL), and two structures after attachment of sialic acid to lactose by the actions of different sialyltransferases, i.e., sialic acid attached by an α2,3-glycosidic linkage to galactose gives 3′-sialyllactose (3′-SL), whereas sialic acid attached by an α2,6-linkage to galactose gives 6′-sialyllactose (6′-SL) [5,21,37]. However, the question concerning which transferases contribute to HMO sialylation remains unanswered. Interestingly, the obtained structures, despite the small structural differences, namely the position of the glycosidic bond with identical qualitative composition for fucosyllactose and sialyllactose, respectively (Figure 1a), show significant functional differences in shaping the gut microbiome and in different levels of protection against fucose- or sialic acid-dependent pathogens, among others [19,32,38,39,40,41,42,43]. The actions of different fucosyltransferases and sialyltransferases allow the identification of over 100 different oligosaccharide structures [21] Depending on the presence of fucose and sialic acid in the oligosaccharide structure, HMOs are divided as follows: fucosylated (neutral) and non-fucosylated (neutral) oligosaccharides and sialylated (acidic) and non-sialylated molecules, respectively. However, some HMOs may contain both a fucose and sialic acid molecule and form an additional subgroup [44].

Concentration and mutual proportions of HMOs are affected mainly by 3 different factors, namely polymorphisms of two fucosyltransferase genes (*FUT2* and *FUT3*), which gives 4 different groups of mothers [20,45], stage of milk maturation [40,46] and the week of delivery [47,48].

Recent studies by Kunz’s group [20] have shown that the total HMO concentration in the milk of mothers who gave birth prematurely did not differ significantly from the milk of mothers who gave birth at term regardless of the stage of milk maturation, namely for colostrum for premature delivery of 8.7 g/L and for term delivery 7.5 g/L, for transitional milk of mothers who gave birth prematurely 8.6 g/L and for term 9.1 g/L, and for mature milk, 8.6 g/L and 8.2 g/L, respectively. The latest research by Austin and coworkers [48] brought the same conclusions. In contrast, the earliest report by Morrow and colleagues [54] showed that the concentration of total HMO in milk of mothers who gave birth prematurely from ten to 23 days of lactation was almost two times lower than in milk of mothers who gave birth at term, in the same period of lactation 3.6 g/L and 6.1 g/L, respectively. 

Mothers, based on their genetic status, can synthesize different sets of oligosaccharides. The most extreme interindividual differences relate to the presence or absence of fucose linked by α1,2- and α1,3/4-glycosidic bonds in free oligosaccharide and glycoproteins, are conditioned by genetic factors (namely active forms of *FUT2* and *FUT3* genes) that affect the secretion status and Lewis blood group antigens [20,37,55,56,57]. Two fucosyltransferases, namely FucT II (encoded by the secretory gene *FUT2*) and FucT III (encoded by the Lewis gene *FUT3*) play a key role in HMOs fucosylation. On this basis, four different groups of mothers are distinguished in whom the profile of fucosylated HMOs is different [20,45]:

1. Secretor (Se+/Le+) – constitutes about 70% of the population,
2. Secretor (Se+/Le−) – constitutes about 9% of the population,
3. Non-secretor (Se−/Le+) – constitutes about 20% of the population,
4. Non-secretor (Se−/Le−) – constitutes about 1% of the population.

Most of the mothers (79%) have an active gene *FUT2* for fucosyltransferase, an enzyme that is responsible for adding Fuc by α1,2 linkage to terminal Gal [58] to form α1,2-fucosylated oligosaccharide structures. In milk of mothers with secretor status, 2′-fucosyllactose (2′-FL) and lacto-N-fucopentaose I (LNFP I) are among the most common [18,59,60]. As was reported by Tonon and coworkers [17] the Se+Le+ phenotype-related differences in abundance of individual HMOs have no effect on newborns’ growth. In contrast, mothers who do not have the functional FucT II enzyme and have “non-secretor” status represent about 21% of women, and produce milk lacking α1,2-fucosylated oligosaccharides such as 2′-FL and LNFP I [20,37].

The total HMO concentration at subsequent stages of lactation is affected by the secretor status of the mother. In milk of non-secretor mothers with positive Lewis status (Le+) the total concentration of HMOs is lower (due to the absence of 2′-FL), but higher abundances of lacto-N-tetraose (LNT), LNFP II, and III and lacto-N-difucohexaose II (LNDFH II) were observed [20]. As was reported by Kunz and coworkers [20] the HMO concentration in the milk of secretor mothers was significantly higher than in the milk of non-secretors, namely 9.67 g/L vs 5.17 g/L for colostrum, 9.47 g/L vs 5.61 g/L for transitional and 8.67 g/L vs 5.54 g/L for mature milk, respectively. 

The data concerning the content of particular fractions, namely fucosylated and/or sialylated, of HMOs are not unequivocal. The earliest studies [61] reported that the proportions of fucosylated and sialylated HMOs in human milk are 60–80% and 10–15%, respectively, and do not differ significantly over milk maturation [61]. Donnovan and Comstock [3] obtained different data for HMO fractions in the milk of mothers who gave birth at term, namely ~35–50% fucosylated, 12–14% sialylated and 42–55% non-fucosylated neutral HMOs. However, the secretor status of the mother is also important. Based on the latest report of Austin and coworkers [48], the α1,2-fucosylated HMOs fraction containing mainly 2′-FL and LNFP-I in milk of mothers who delivered prematurely was lower than in term milk due to the not fully active *FUT2*. Additionally, the level and composition of particular fucosylated HMOs is significantly different in milk of non-secretor mothers [20], especially during early lactation [40].

Nevertheless, in the case of HMOs, the question of what the physiological range is still remains unanswered [62]. Additionally, due to the structural complexity of HMOs, as well as the lack of a wide range of standards and the use of different sophisticated methods for qualitative and quantitative determination of total and individual milk oligosaccharides, the available data vary significantly and continue to pose a major challenge [35].

Moreover, the latest multicenter studies have shown that the HMO profile in breast milk can be influenced by environmental factors [62]. The concentrations of individual fractions, both fucosylated and sialylated, and even individual HMOs, can differ geographically. Almost all concentrations of individual HMO structures were affected. The concentration of individual fucosylated oligosaccharides such as 3-FL was more than four times higher in mother’s milk in Sweden than in mother’s milk collected in rural Gambia [62]. However, to confirm this hypothesis, targeted genomic analyses are needed to determine whether these differences are due at least partly to genetic variation.

Additionally, the presence of some individual HMOs has an impact on the level of other oligosaccharide structures. An observational, single-center, longitudinal cohort study showed that 2′-FL is engaged in positive and negative ‘co-regulation’ of non-fucosylated oligosaccharides, namely LNnT and LNT, respectively [46].

## 4. Fucosylation of Human Milk Glycoproteins

Human milk glycoproteins (HMGs) are an important component of human milk and actively participate in ensuring the proper development and protection of the immunologically immature newborn. However, except lactoferrin and S-IgA, they have been characterized in much less detail compared to HMOs [6,7,40,63].

For HMGs, the most common feature is fucose attached by α1,6 linkage to GlcNAc of glycoprotein N-glycans, the so-called “core” fucose. The addition is done due to the α1,6-fucosyltransferase encoded by the *FUT8* gene. The presence of core fucose is characteristic for glycoproteins produced by liver cells and is particularly important for biological functions of proteins [8,60]. However, so far there are no reports concerning the possible differences in core fucosylation of milk glycoproteins caused by genetic factors. The cooperation of the set of fucosyltransferases and other enzymes involved in synthesis and posttranslational modification of the glycan part of glycoproteins within alveolar cells of mammary gland is responsible for a huge variety of individual HMOs and HMGs; however, up to now the presence of Fuc in the glycan part of milk glycolipids has not been reported [7,64,65,66].

Most of the studies focus on two major glycoproteins, namely LF and S-IgA, whose concentrations in milk are sufficient for isolation and structural analysis using advanced methods [37,67]. The glycosylation level of human milk lactoferrin from five donors during the first 10 weeks of lactation was characterized by a decrease in the second week followed by an increase in total glycosylation thereafter. Moreover, an increase in fucosylation degree was observed with the progression of lactation. The observed trends overlap with the changes in gene expression of enzymes involved in glycosylation, such as a decrease of gene expression for the oligosaccharyltransferase complex in the second week of lactation [67]. However, up to now, no reports are available clarifying the impact of changes in the glycosylation profile of LF on the biological function of this glycoprotein.

On the other hand, the analysis of glycosylation of S-IgA showed that both O-glycans of heavy chain and N-glycans of the secretory fragment (SC) contain fucosylated (Fuc(α1,3/4)GlcNAc, Fuc(α1,2)Gal) and/or sialylated (Neu5Ac(α2,3/6)GlcNAc) glycotopes, which can be additional bacterial binding sites and are among the elements of innate immunity [4]. The presence of N- and O-glycans on the S-IgA molecule, in addition to the four antigen binding sites (Fabs), is suggested to be a link between innate and acquired immunity [4].

It is interesting that some glycoproteins, namely IgG [68], AGP [29], and FN [31], which are present in both human milk and mother’s plasma, have quite different glycosylation patterns. The changes in glycosylation are the net result of local biosynthesis of those glycoproteins by hormonally regulated alveolar cells of the mammary gland [57,69]. The fucosylation profile of the second most abundant immunoglobulin in human milk, namely IgG, was shown to differ qualitatively and quantitatively from maternal IgG [68]. Moreover, the pattern of α1,2-, α1,6- and α1,3-fucosylated glycotopes of milk IgG was lactation stage and gestational week dependent.

Detailed studies have revealed that, during progression of lactation, the fucosylation degree of all human skim milk glycoprotein differs in relation to the type of fucose linkage to the oligosaccharide part of glycoprotein, as well as to the analyzed glycoprotein [29,31,68,69]. The overall α1,2- and α1,6-fucosylation levels of HMGs for mature skim milk were 42% and 49% of those observed for early colostrum, respectively, while α1,3-fucosylation of HMGs remained at an unchanged low level [70]. Interestingly, the level of major α1,2-fucosylated HMGs overlaps with the trend reported for the simplest fucosylated HMOs, namely 2′-FL [46,48], during milk maturation.

The second trend of HMG analysis is focused on determination of the glycan profiles of milk glycoproteins in relation to the type of glycosidic bond, namely N- and O-linked glycans. For methodological reasons, previous analyses concerned N-glycans only. The first studies by Nwosu and coworkers [71] showed that 25 out of 38 identified N-glycans attached to HMGs are fucosylated, which makes up to 75% of all, in contrast to bovine milk N-glycans, only 31% of which are fuco sylated. However, in recent years, another global approach has emerged for the analysis of glycans attached to milk glycoproteins, namely the analysis of the profile of all N- and O-linked glycans (Figure 1b,c) [52,53]. Similarly to HMOs, the glycosylation profile of HMGs is related to the milk maturation stages, but the opposite trends were observed depending on the type of glycosidic bonds. During milk maturation, fucosylated N-glycans of HMGs gradually decreased, but in the same lactation period, an increase in fucosylated O-glycans is observed [53]. In parallel, more detailed studies on individual HMGs revealed that although the concentration of major milk glycoprotein decreases with lactation progression, the attached glycans elicited independent quantitative changes in their pattern. During milk maturation, the amount of fucosylated glycans of lactoferrin and S-IgA significantly increases [52]. Considering the fact that the oligosaccharide chain bound to glycoproteins might be elongated by different fucosylated or sialylated glycotopes, the attached glycans might have different susceptibility to proteolytic cleavage, pathogen binding, prebiotic and health effects in newborns and infants [6]. However, the knowledge concerning the impact of oligosaccharide part of human milk glycoproteins in the proper development of the neonate needs to be elucidated.

## 5. Maternal Morbidities and Their Impact on Fucosylation of Dietary HMOs and HMGs

The scientific data concerning human milk glycomics in relation to maternal disease is fragmentary. However, it was reported that diabetes mellitus [72,73,74], overweight [75,76] or hypertension [77] of the lactating mother has an impact on the nutritional composition of milk. A summary of the main trends observed in the nutritional composition of mother’s milk was presented by Amaral and coworkers [78] as well as the systematic review by Peila and coworkers [79].

In contrast to the main components of mother’s milk, the impact of maternal morbidities on the glycosylation and fucosylation status is much less characterized. However, the detailed characteristics of the glycosylation profile, including fucosylation and sialylation of HMOs and glycoproteins in relation to the pathophysiological status of the mother, is particularly important for postnatal care. The first report in this field showed [80] that the fucosylation degree of S-IgA and LF from milk of mothers with gestational diabetes elicited a significant decrease and increase, respectively, in relation to fucosylated N-glycans of S-IgA and LF from milk of healthy mothers [81], as a consequence of maternal glucose dysregulation. Moreover, the presence of maternal infection has an impact on the fucosylation profile of milk IgG, namely lower and higher levels of α1,2- and α1,3-fucosylated glycotopes, respectively, were observed [68]. In light of the significant role of the oligosaccharide part of glycoproteins in proper function of molecules, the changes in their glycosylation pattern might be crucial for biological functions. So far, the impact of the changes in glycosylation profile of human milk glycoproteins on their function and on the newborns/infants has not been studied.

The unprecedented recent study of breastfeeding Brazilian women showed that maternal and infant factors might influence concentration of HMOs in some SeLe group. The novel associations of infant’s sex as well as maternal allergic disease with the mother’s secretor phenotype were demonstrated [17]. As reported by the authors, mothers of boys had a significantly lower level of 2′-FL in their milk than mothers of girls (2.3 and 4.8 g/L, respectively) and on the other hand, mothers of girls had lower LNH concentrations than mothers of boys (0.02 and 0.10 g/L, respectively), as well as LNT+LNnT (0.17 and 0.54 g/L, respectively) and total neutral core HMOs (0.20 and 0.67 g/L, respectively), however, for Se+Le− mothers only. Moreover, it was shown that the concentration of difucosyl-para-lacto-N-neohexaose (DFpLNnH) was higher in milk from secretor mothers (Se+Le+ and Se+Le−) with allergic disease (asthma, rhinitis or eczema) than in milk from mothers without allergic disease. Interestingly, considering only secretor phenotype of mothers, no differences in concentrations of DFpLNnH or another HMO between analyzed groups (Se+Le+, Se−Le+, Se+Le−) were observed. These results obtained by Tonon and coworkers [17] are not in line with the data reported earlier by Sjögren and coworkers [81]; however, their study was based on a smaller cohort group and the results for nine neutral HMOs (but not DFpLNnH) were considered depending on the mother’s health status only (allergic and non-allergic mothers).

It is also worth mentioning that some conditions of mothers have a decisive impact on the mode of delivery which is particularly important for the children of mothers with non-secretory status. Recent studies have shown [82] that coexisting non-secretor status of the mother and cesarean delivery (C-section) deeply change the newborn’s microbiome. As speculated by the authors [82], the determination of secretor status of mothers giving birth by C-section allows the identification of infants with special needs who in parallel with mother’s milk feeding should be supplemented with bifidogenic α1, 2-fucosylated HMOs and bifidobacteria.

## 6. Shaping of Infant’s Gut Microbiome by Dietary HMOs

In contrast to the main milk sugar lactose, which is broken down in the small intestine by lactase into simple sugars, HMOs and the glycan part of glycoproteins are not digested and pass through the gastrointestinal tract of breastfed newborns [83,84]. In the lumen of the intestine, non-digestible milk oligosaccharides contribute to human health by shaping the newborn’s microbiome and inhibit colonization by pathogenic bacteria [5,9,84,85,86]. However, some species, namely bifidobacteria, are able to utilize HMOs and glycans of milk glycoconjugates [85,87,88,89]. Moreover, the glycans attached to milk glycoproteins may elicit their biological function in the infant’s gut since it was shown that lactoferrin and its fragments were identified in the stool of breastfed infants [90].

**Figure 2:**
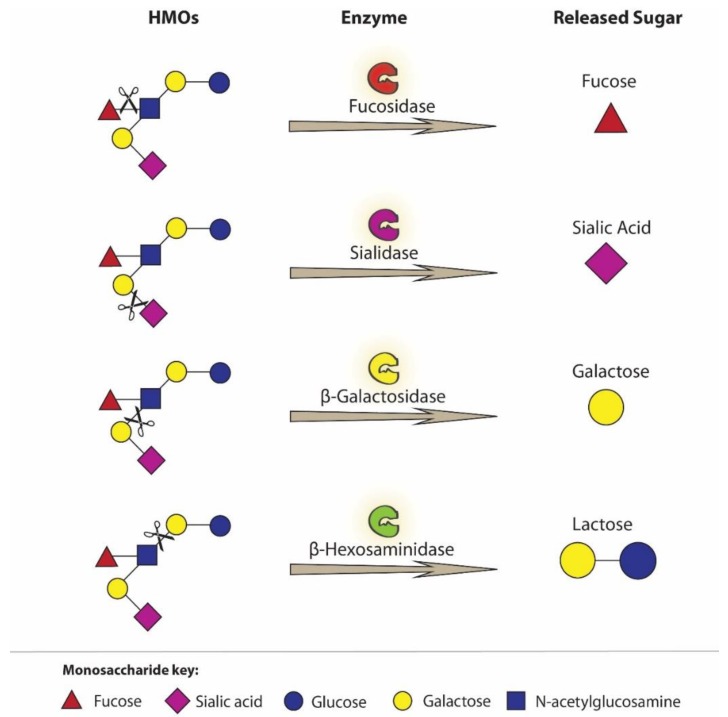
Bifidobacteria species present in infant gut encoding different glycosidases, responsible for cleaving individual monosaccharide units from HMOs [85,91,92,93]. HMOs, human milk oligosaccharides.

*Bifidobacterium* and *Lactobacillus* spp. present in the digestive tract of newborns and infants differ in their ability to use and/or digest HMOs. In comparison with *Lactobacillus gasseri*, *Bifidobacterium infantis* has an excellent ability to digest HMOs [91,92]. The genome of *B. infantis* encodes 24 different glycosidases, responsible for cleaving individual monosaccharide units from oligosaccharides, including 2α-sialidase and 5α-L-fucosidase (Figure 2) [85,91,92,93].

It was reported [85] that HMOs with Fuc linkage by α1,2-glycosidic bonds promote the growth of *Bifidobacterium longum* subsp., *B. bifidum* subsp., and *B. breve* spp., which is connected with ability of these bacteria to hydrolyze 2′-fucosylated HMOs. In addition, *Bifidobacterium bifidum* may release other monosaccharides from HMOs, but cannot use monosaccharides such as fucose, sialic acid and N-acetylglucosamine [94]. On the other hand, *Bifidobacterium breve* cannot “release” individual monosaccharides from HMOs, but in turn has the ability to use them if they are present in the free form [91,95].

**Figure 3:**
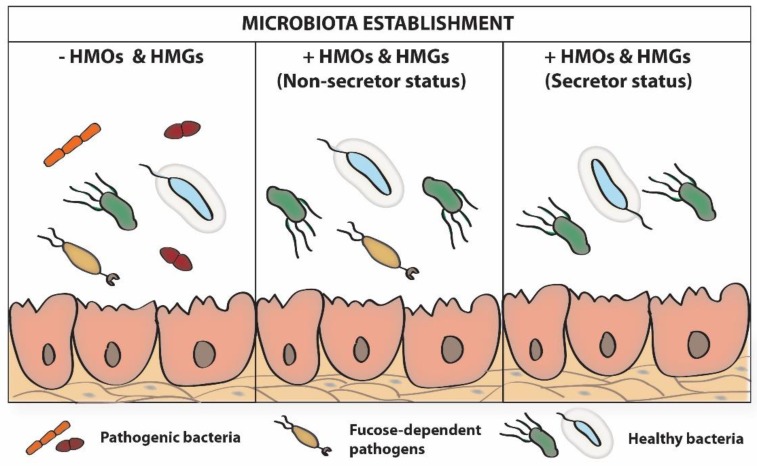
The impact of maternal secretor status on infant’s microbiota establishment [96,97]. HMOs, human milk oligosaccharides; HMGs, human milk glycoproteins.

In line with the above, it is also important to pay attention to an additional important factor, the interrelationships between the various components of the infant’s intestinal microbiome related to the type of feeding. Moreover, among breasted infants, differences in microbiome related to the secretor/non-secretor status of mothers are observed (Figure 3) [96,97]. Additionally, during each breastfeeding, subsequent “doses” of bacteria, including *Staphylococcus*, *Streptococcus*, *Bifidobacterium*, and *Lactobacillus* [98], present in maternal milk are delivered. So, the breastfed infants in contrast to the formula-fed infants have contact with another set of bacteria, as well as oligosaccharides and glycoproteins, which all have an impact on significant differences in colonization and maturation of the infant’s gut [96,97,99].

Breastfeeding shapes the intestinal microflora of newborns and infants, both directly through the “exposure” of the newborn to the microflora present in breast milk, and indirectly, through breast milk factors such as HMOs, which are a key influence on bacterial growth and metabolism [100]. In this aspect, HMOs participate in formation of a healthy microbiome and thus seem to be a promising factor in preventing allergies [101]. The latest study by Lawson and coworkers [102] shows that bifidobacteria strains present in the infant ecosystem share delivered nutrients to maximize their utilization, including HMOs, from the diet. These data suggest that bifidobacteria-based therapies may be useful for neonatal health-disease balance to promote infant health.

It should also be emphasized that not only the free milk oligosaccharides can be utilized by probiotic bacteria. It was shown that EndoBI-1 (endo-β-N-acetylglucosaminidase) of bifidobacteria can cut off the N-linked glycans from bovine colostrum glycoproteins and the released N-glycans may give rise to the selective growth of *Bifidobacterium.* However, the bifidobacteria species differ in their consumption. In detail, *B. lactis* digested only 11% of neutral N-glycans, in contrast to *B. infantis*, able to digest 73% of neutral and 92% of sialylated N-glycans [88].

An in vitro study based on a mouse model using Chinese mothers’ milk glycoproteins N-glycans with core fucosylation showed that such structures have an impact on the growth of *Bifidobacterium* and *Lactobacillus* and the fucose metabolites may initiate via B cell receptor the activation of infants’ B cells [8]. In line with the above, α1,6-fucosylated N-glycans attached to maternal milk glycoproteins may be considered as potential natural prebiotics, supplementary to HMOs, important for newborn and infant nutrition [8].

## 7. Dietary Fucosylated HMOs and HMGs Have Anti-Adhesive Properties

Mucosal surfaces are the main contact point between infants and pathogens present in the external environment. The proper protective function of membranes is related, among other factors, to the presence of particular oligosaccharide structures containing fucosylated and/or sialylated glycotopes involved in biological recognition processes between cells. Differences in the amount and profile of mucosal glycans may modulate susceptibility to infection [103,104].

**Figure 4:**
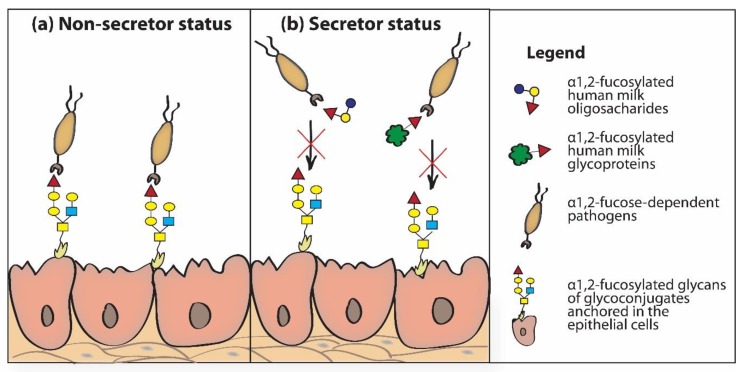
The impact of non-secretor (**a**) and secretor (**b**) status of mother on inhibition of α1,2-fucose dependent pathogen adhesion to epithelial cells of the newborn’s/infant’s gastrointestinal tract [117,118].

The mechanism of anti-bacterial and anti-viral activity of fucosylated HMOs is based on the structural similarity of individual HMOs to sugar chains of glycoproteins present on the surface of neonatal/infant epithelial cells. Thanks to this, HMOs “mimic” surface glycans of epithelial cells [5,9,50,86]. Soluble fucosylated human milk oligosaccharides “passing” through the newborn’s digestive tract “rinse” the epithelial cells of the throat, esophagus and intestines of the newborn and can be recognized and bound by (1) fucose-specific lectin receptors present on the surface of host epithelial cells and/or via (2) lectin receptors of fucose-dependent bacteria. In both cases, fucosylated HMOs, as “decoy inhibitors”, participate in blocking lectin receptors. Bacterial lectin receptors blocked by HMOs cannot participate in the reaction of recognizing glycotopes present on the surface of host cells, which prevents their adhesion and colonization, and formed bacterial-HMO complexes are removed with feces [5,30,33,105,106]. Inhibitory effects of HMOs in adhesion to host tissues have been demonstrated for fucose-dependent pathogens, such as *Campylobacter jejuni* [38], enteropathogenic *Escherichia coli* [107,108], *Listeria monocytogenes* [109], *Pseudomonas aeruginosa* [108], *Helicobacter pylori* [110], *Vibrio cholerae* [111,112], some viruses (Noroviruses) [113,114,115], and human immunodeficiency virus (HIV) [116], among others. The participation of α1,2-fucosylated oligosaccharides and glycoproteins of human milk in inhibition of α1,2-fucose dependent pathogen adhesion to epithelial cells of the newborn’s/infant’s gastrointestinal tract depends on the secretor status of the mother. The lack of the α1,2-fucosylated HMOs and soluble α1,2-fucosylated HMGs in non-secretor mothers’ milk is responsible for absence of inhibition of the adhesion of α1,2-fucose dependent pathogens to the epithelial host cells (Figure 4a). In the presence of α1,2-fucosylated HMOs and soluble α1,2-fucosylated HMGs in secretor mothers milk the adhesion of α1,2-fucose dependent pathogens to the epithelial host cells is blocked (Figure 4b) [117].

It has been shown that 2′-FL and additionally 3′-SL can reduce the incidence of viral infections caused by respiratory syncytial virus (RSV) in vitro by significantly reducing RSV viral load and the level of cytokines in the airway epithelium [119]. Moreover, the latest research shows that HMOs reduce the infectivity of human rotaviruses in vitro; however, the maximum reduction for rotavirus G1P serotype was observed with 2′-FL, whereas for rotavirus G2P serotype it was observed with 3′-SL and 6′-SL [120]. In a mouse model, it was shown that HMOs increase the expression of mucins on the surface of intestinal epithelial cells, which translates into a reduction of adhesion of enteric pathogens [121]. In addition, fucosylated HMOs, due to the properties of lowering the rolling and adhesion of leukocytes to endothelial cells, can participate in silencing immunological processes, such as the ability of phagocytosis and the production of reactive oxygen species, which are important in the development of necrotizing enterocolitis [122,123,124,125]. Similar to HMOs, glycans attached to HMGs also elicited anti-adhesive properties. As was reported by Barboza and coworkers [67] based on the in vitro invasion assay, N-glycans derived from human milk lactoferrin blocked the invasion of Caco2 intestinal epithelial cells by *Salmonella.*

## 8. Dietary Fucosylated HMOs in Infant’s Blood

Due to the unique and complex structures, HMOs are resistant to hydrolysis in the gastrointestinal tract and digestion by enzymes [126]. However, the epithelial barrier of the newborn/infant intestine can be crossed by both neutral and acidic intact HMOs, although only for neutral HMOs has active transport been confirmed [127]. Ingested HMOs can be absorbed in the small intestine of a newborn and remain intact in its bloodstream even for several hours and then are removed with the urine. However, the level of HMOs in infant’s plasma and urine was very low in relation to milk and was 0.1% and 4% of the milk level, respectively [128]. Ruhaak and coworkers [129] reported the presence of free oligosaccharides, similar to those present in mother’s milk, in infant’s plasma; nevertheless, the profile was different depending on the type of feeding, namely breastfed or formula-fed. The typically detected oligosaccharides included all types of structure, namely neutral LNT, fucosylated 2′-FL, LDFP, LNFT, and sialylated 3′-SL, 6′-SL, 3′-SLN, and 6′-SLN. However, 2′-FL was present in blood even if the infant was breastfed partially but was absent in blood of infants exclusively formula-fed.

Moreover, the concentration of the simplest fucosylated oligosaccharides such as 2′-FL and 3-FL in infant’s plasma are strongly associated with the levels in mother’s milk [128]. The concentration of the most abundant oligosaccharide, 2′-FL, in the blood of newborns reaches a concentration of 1.5 mg/L. HMOs are then removed from the infant’s circulation along with urine, in which 2′-FL is concentrated and reached 100 mg/L [5,124,130]. The oligosaccharides present in the urine can, similarly as in the gastrointestinal tract, prevent the adhesion of pathogens to the epithelial cells of the urinary tract and thus actively participate in protecting infants against pathogens causing urinary tract infections [5,124,130].

## 9. Dietary Fucosylated HMOs as a Substrate for Synthesis of New Structures

HMOs present in the infant’s bloodstream are important for normal brain development, namely fucosylated HMOs are suggested to be an additional source of fucose for the synthesis of new glycoconjugates, which play an important role in the proper development and functioning of the central nervous system, including impulse transmission between neurons [5,124]. Promising data were obtained on an animal model [131]. It has been shown that 2′-FL oral administration significantly enhances cognitive processes and memory in supplemented rat pups. Moreover, 2′-FL given in the period of lactation raised cognitive skills during childhood as well as in later life, at age 1 year.

The latest research of mother-infant pairs suggested that the frequency of breastfeeding at 1 month due to the higher exposure to mother’s milk 2′-FL promotes cognitive development of newborns, evaluated at 24 months with the Bayley-III Scale [132]. However, the same effect was not observed for 6-month-old newborns. In the context of these studies, the question arises, which we still do not know the answer to, whether newborns and infants fed milk of mothers with the non-secretor status are more “at risk of disorders” due to the lack of dietary 2′-FL.

## 10. Dietary Fucosylated HMOs and HMG Glycans as Immunomodulators

HMOs can also modulate the functioning of the infant’s immature immune system at the cellular level due to the structural similarity between oligosaccharides and glycotopes of glycoprotein. HMOs can be recognized by lectin type receptors common in biological recognition processes. The potential receptors of HMOs in the immune system are C-type lectins, galectins, selectins and siglectins, which differ in their specificity against sugar ligands [130,133]. With the exception of siglectins specific to sialylated structures, the lectin receptors are able to recognize different fucosylated structures, namely 2′-FL, 3-FL, LNFP-III, LNFP-IV, LNDFH-I for C-type lectin [133], NFP I, LNFP II, LNFP III, LNDFH, FucLac for galectins [134] and sialyl-Lewis x (sLewis x) for selectins [135], among others.

Selectins participate in interactions which are crucial for cell adhesion and migration, as the basis for pathological processes such as inflammation and cancer, but also play an essential role in physiological processes such as leukocyte homing [136,137]. During inflammatory processes, E and P selectins present on the surface of endothelial cells recognize and participate in interactions with sLewis x glycotopes, part of the glycoconjugates on the surface of leukocytes, which are one of the elements involved in the process of leukocyte extravasation and mucosal infiltration [22,124]. Similarly, some human milk oligosaccharides, whose chemical structures are similar to fucosylated and sialylated sLewis x and sLewis a antigens, may be recognized by immune receptors of infants and thus may be involved in modulating selectin-mediated cell adhesion in breastfed infants as selectin ligands [22,136,138].

The first experimental data suggesting that HMOs have the ability to “interfere” with the process of leukocyte rolling and potentially reduce their extravasation were presented by Rudloff and colleagues [135]. Based on previous experiments [135], Bode and Jantscher-Krenn [22] speculate that 3-FL present in human milk and more structurally complex HMO molecules that in their structure contain more than one sLewis x glycotope, which enables multivalent binding to selectins, may participate in inhibition of rolling and subsequent leukocyte adhesion [22,135].

## 11. Dietary Fucosylated HMOs and Development of Allergy

The protective role of breastfeeding against the development of allergic sensitization and allergic disease is not clear. The impact of individual differences between mothers in milk composition such as HMOs, glycoproteins, metabolites, hormones, and also intestinal microflora, has an import role in the considerable heterogeneity of obtained results. The association between breastfeeding/human milk composition and the development of allergic diseases was discussed in detail in a review by Munblit and coworkers [139]. Moreover, the protective effect of HMOs on infant allergy development was reported [46,81,139,140]. According to Järvinen and coworkers [141], the manifestation of an allergic disease is related to individual predisposition determined by genetic factors as well as to diet and even intestinal microflora. Moreover, the type of feeding together with the mode of delivery as well as exposure to antibiotics are the main factors of microbiome shaping [142].

The first prospective study concerning the relations between allergy and neutral HMO composition, namely the development of atopic symptoms in children up to 18 months of age in relation to maternal allergy and environmental factors, was reported by Sjögren and coworkers [81]. However, the level of individual neutral fucosylated HMOs such as 2′-FL, 3-FL, LNDFH, LNFP I, LNFP II, LNFP III, and LDFT in colostrum of non-allergic and allergic mothers did not show any differences. Moreover, the highest, although not significantly, total concentration of HMOs has no impact on development of allergy. Another study showed that infants feeding with maternal milk with a low level of fucosylated oligosaccharide, namely lacto-N-fucopentaose (LNFP) III, were more likely to develop a cow’s milk allergy than infants fed with maternal milk with higher concentration, although the possible impact of antibodies, cytokines, or exosomes, cannot be excluded [143].

Since the composition of HMOs is affected by secretor status of the mother, the question arises whether it can affect the development of allergies later in life. The preliminary observations of infants born by C-section with high hereditary allergy risk reported by Sprenger and coworkers [46] showed that the presence of α1,2-fucosylated HMOs, characteristic for secretor mothers, probably has an impact on lowering the risk of allergy of 2-year-old children. Unfortunately, this effect was no longer visible in children aged 5 years. Additionally, in colostrum of mothers giving birth by C-section significantly lower concentrations of fucosylated oligosaccharides, namely 2′-FL, LNFP II, LNFP III, LNnDFH, were observed [144].

## 12. The Impact of Dietary Fucosylated HMOs on Development of Immune Tolerance

Dendritic cells (DCs) equipped with different types of lectin receptors are the primary cells which take part in a wide range of immune responses. DCs present in the gastrointestinal tract of neonates, due to the expression of dendritic cell-specific intercellular adhesion molecule-3-grabbing non-integrin (DC-SIGN), can recognize and bind human milk glycans containing terminal fucose in their structure [145]. Subsequent studies have shown that DC-SIGN on the surface of DCs binds to fucosylated milk oligosaccharides, namely 2′-FL and 3-FL, and such recognition is suggested to participate in modulation of the immune response and in ensuring immune homeostasis of the breastfed newborn [133]. Moreover, human milk 2′-FL participates in attenuating LPS-induced inflammation through modulation of CD14 expression in human enterocytes [146].

Recent research [147] showed that HMOs induce the semi-maturation of DCs, which play a key role in regulating the immune response, including specializing in antigen presentation and T cell differentiation. In addition, HMOs increase IL-10, IL-27 and IL-6 levels, but have no effect on IL-12p70 and tumor necrosis factor α (TNF-α) levels. Consequently, under the influence of HMOs, human monocyte-derived DCs promoted the production of regulatory lymphocytes (Tregs) from CD4 + T cells [147]. The regulatory role of HMOs appears to be based on their interactions with lectin receptors on DCs, such as the well-characterized pathogen recognition receptors TLR4 and DC-SIGN among others, essential in regulation of host–microbe interaction. In line with the above, it is suggested that HMOs contain tolerogenic molecules, affecting human DCs, and in such a way take part in shaping the immature immune system of newborns and additionally in modulation of bacteria-independent inflammatory events.

## 13. Dietary Fucosylated HMOs and HMGs in Donor Milk

Milk obtained from donor mothers and deposited in milk banks before being given to newborns must undergo an appropriate procedure which aims to remove potentially pathogenic microorganisms while maintaining the highest possible biological activity [148]. Regardless of the methods of pasteurization of mother’s milk, which to various degrees destroy or partially inactivate many bioactive milk components, HMO structures remain intact after Holder pasteurization and freeze-drying [149] as well as after simulated flash heating pasteurization [150], so they are still able to elicit as wide a range of biological functions as non-treated HMOs. However, the storage length of donor milk in frozen form without affecting HMOs requires additional and more accurate analyses. Nevertheless, some techniques may affect the milk HMO profile. Studies using retort sterilization of donor milk have shown that this treatment of milk reduces both total and fucosylated HMOs in comparison to milk samples after Holder and vat pasteurization [151].

Another important factor in the context of fucosylated HMOs is individual differences among breastfeeding mothers not related to secretor status [48,62,144]. Moreover, the results regarding differences between donor’s milk deposited in milk banks and the mother’s own milk seem surprising. In donor milk, the concentration of HMOs as well as individual oligosaccharides such as LNT, lacto-N-neotetraose (LNnT), LNFP I, and disialyllacto-N-tetraose (DSLNT) were lower than in mothers’ own milk. In contrast, the level of 3-FL and 3′-SL was higher in comparison to the mother’s own milk [152]. Nevertheless, more detailed studies on larger study cohorts and with additional consideration of the effect of genetic factors responsible for secretor status are needed.

Unfortunately, so far based on searching via PubMed no data are available concerning the glycosylation profile of donor milk glycoproteins as well as the effect of methods of pasteurization on the structures of N- and O-glycans attached to HMOs.

## 14. Fucosylated HMOs and Bovine Glycoproteins in Infant Formula

Thanks to intensive development in the field of biotechnology (advanced biotechnological and/or chemical methods of oligosaccharides synthesis) and the possibility of obtaining large quantities of bioactive compounds on an industrial scale, there were efforts to enrich the artificial milk mixtures of certain oligosaccharides structures identical as in human milk [3,5,21,23,138,153,154,155,156]. Enrichment of bovine-based infant formula with fucosylated oligosaccharides is necessary since the concentration and composition of cow’s milk oligosaccharides are radically different from those of human milk (Table 2).

Presently, due to having the highest concentration among human milk oligosaccharides, 2′-FL is the best characterized and tested oligosaccharide which is added to infant’s formula [15]. By 2019, nearly 200 US patents on adding 2′-FL as a food ingredient had been reported [157]. Recently, two detailed reviews concerning the addition of individual HMOs, namely fucosylated 2′-FL and non-fucosylated lacto-N-neotetraose (LNnT), to different infant formulas by Reverri and coworkers [158] and by Vandenplas and coworkers [15], respectively, were published.

Already in 2015 the European Food Safety Authority (EFSA) [159] stated that 2′-FL is safe for infants when added to formula milk in a concentration up to 1.2 g/L alone or in combination with LNnT at concentrations up to 1.2 g/L and up to 0.6 g/L, respectively. In line with the above, the concentration of 2′-FL added to formula milk is lower than in mother’s milk (2′-FL in human milk is the most abundant oligosaccharide with the average concentration 2.7 g/L, range 1.88–4.9 g/L) [3]; however, the concentration of 2′-FL decreased with milk maturation [46,48]. Despite this, the scientific reports [15,160] suggest that the supplementation of formula milk with 2′-FL seems to be well tolerated, and has an impact on proper growth and development and well-being of newborns and infants.

In a randomized controlled trial [161] it was shown that the addition of 2′-FL (at two different amounts, namely 0.2 and 1 g) to infant formula led to lowering of the level of IL-1α, IL-1β, IL-6, and (TNF-α) in comparison to infant formula without 2′-FL. Moreover, the level of inflammatory cytokines in serum of infants fed with formula supplemented with 2′-FL was at the same level as for breastfed infants.

Salli and coworkers [162], using a semi-continuous colon simulator, evaluated the impact of 2′-FL, galacto-oligosaccharide (GOS) and lactose on composition of the infant microbiota and microbial metabolites. The obtained data showed that the discrete changes in microbiota associated with the metabolism of 2′-FL resulted in the intermediate production of short chain fatty acids and lower production of acetate and lactate in comparison to the control. Moreover, the authors stated that fermentation of 2′-FL requires more specific microbial activity in comparison to fermentation of lactose or GOS [162].

The latest research on a rat model showed that not only is 2′-FL supplementation safe, but also the mixture of the five simplest fucosylated and sialylated structures, i.e., 2′-FL, 3-FL, 3′-SL, 6′-SL, and LNT, was not genotoxic [163]. Moreover, in the repeated-dose study, an adverse effect was not observed. The obtained results provide the first relevant data which may be useful for developing new artificial milk mixtures for human newborns and infants. However, from the rational and economic point of view, obtaining milk on a large scale from donor mothers should be taken into consideration.

Moreover, recent reports [16,157] show that interest in the addition of fucosylated oligosaccharides, mainly 2′-FL, is growing not only for infant formula, but also in highly specialized food preparations that can be used in the clinic and which are extremely important for the health of the general public. Additionally, to meet the needs, new techniques have been developed that allow the determination of fucosylated milk oligosaccharides such as 2′-FL and 3-FL not only in breast milk, but also to control levels in commercially available infant formulas [164]. Such a modern analytical approach also has potential application to carry out stability tests in various food applications.

In the context of mixtures based on cow’s milk, differences regarding not only oligosaccharides and proteins but also the glycosylation profile of milk glycoproteins should be taken into consideration [165,166]. The comparison of the N-glycan pattern of the whey milk fraction [167] and milk fat globule membrane [168] of both human and bovine colostrum and mature milk elicited significant qualitative and quantitative differences and the N-glycosylation profile was strongly affected by the stage of lactation. Only 12% of N-linked glycans were observed in both human and bovine colostrum and mature milk. Moreover, detailed analysis indicated that the profile of N-glycoproteins with immunological properties was different. The differences in structure of N-glycans also has an impact on anti-pathogenic properties of the oligosaccharide part of human milk glycoproteins. According to Wang and coworkers [165] the N-glycans isolated from glycoproteins of human and bovine milk differ significantly in anti-pathogenic activity against *Escherichia coli*, *Listeria monocytogenes*, *Salmonella typhimurium*, *Shigella sonnei* and *Staphylococcus aureus*. Higher anti-pathogenic activity is strongly related to the level of fucosylated N-glycans and nearly disappeared after removing fucose from glycans [165].

## 15. Fucosylated HMOs Are Present not only in Human Milk

In the light of recent reports, newborns already have contact with free oligosaccharides during the prenatal period of their life. Last year, Wise and coworkers [169] published the first study indicating that free oligosaccharides are also present in the urine of mothers and amniotic fluid at birth, but at a lower level than in four-day colostrum. The presence of 2′-FL, 3-FL, difucosyllactose (DFL), and 6′-SL was demonstrated, but their profile differed from that for colostrum. Based on the foregoing, the developing fetus has “contact” with HMOs during at least the latest stage of pregnancy. This phenomenon justifies future research to investigate the direct and long-term consequences for the health and development of the fetus and infants. Moreover, HMOs were also found in maternal serum during pregnancy and their concentration and composition differed depending on the gestational age and secretory status of mothers [170]. The total HMO concentration increased with gestational age and changed from a mainly sialylated HMO profile at 10-14 weeks of gestation to a more balanced ratio of fucosylated to sialylated HMOs at 30–35 weeks of gestation, mainly due to a significant increase in 2′-FL. Additionally, it is speculated that maternal body composition affects the oligosaccharide profile during pregnancy [170]. Moreover, the level and profile of individual HMOs is similar for maternal and cord blood, which confirms the placental transport of oligosaccharides from mother to fetus [171]. Among individual oligosaccharides, the strongest relationship in concentration was observed for two fucosylated oligosaccharides, namely for 2′-FL and LDFT. Using an ex vivo placental perfusion model, it was shown that 22% of maternal 2′-FL reached the fetal circulation, but without achieving a balance [171].

## 16. Non-Human Fucosylated Milk Oligosaccharides

The number of studies concerning the oligosaccharide structures in mammalian milk has increased rapidly in the last few years. Due to the advanced technologies adopted for sugar analysis such as mass spectra, a huge number of structures have been identified and characterized. Moreover, significant differences in chemical structure of free milk oligosaccharides between species, both qualitative and quantitative, were described in detail [153,172,173,174,175]. Such extraordinary diversity is suggested to have a direct impact on eliciting a wide range of biological functions.

Free oligosaccharides present in domestic mammals’ milk are different from the human milk oligosaccharide profile, and their concentrations are approximately 10–100 times lower than in human milk [15,16,17,174,176,177,178] regardless of the lactation stage. In addition to the total concentration of oligosaccharides, the most spectacular differences relate to the presence of fucosylated structures, characteristic for human milk (Table 2). It is speculated that oligosaccharides of human milk and bovine milk (BMOs), due to the significant differences in concentration and structures, show different levels of protection against human pathogens [7].

## 17. Conclusions

During breastfeeding, mother’s milk provides newborns and infants with biologically active components necessary for proper growth and development, as well as immunomodulatory molecules, including fucosylated HMOs and HMGs, which support their immature immune system. Dietary fucosylated oligosaccharides and glycoproteins delivered with mother’s milk benefit the newborns and infants. Based on a wide range of scientific reports, they participate in the protection against fucose-dependent pathogens, modulate the infant’s microbiome and support the development and maturation of the immature immune system of newborns and infants, including at the cellular level. In this approach, dietary milk oligosaccharides are classified as part of the innate immunity passed to offspring along with breast milk. Nevertheless, due to genetic factors, about 20% of mothers do not provide their infants with dietary 2′-FL and other α1,2-fucosylated oligosaccharides and glycans of glycoproteins, even though they breastfeed them. This important fact should be taken into consideration, and this group of newborns should be supported with supplementary supervision. The lack of such α1,2-fucosylated structures in maternal milk may have important implications for a wide range of aspects of infants’ well-being and healthcare. Moreover, one can speculate that in clinically justified situations, it may be the basis for considering nutritional intervention in infants of mothers with non-secretory status.

## Figures and Tables

**Table 1:** Search strategies and terms used to identify studies for this review.

Search Terms Used to Identify Studies on Human Milk	Search Terms Used to Identify Factors Associated with Fucosylated Glycans Content	Search Terms Used to Identify Factors Associated with Infant Well-Being
human milk AND fucosylation OR fucose AND oligosaccharides OR glycoproteins	secretor status	2′-fucosyllactose OR 2′-FL
human lactation AND fucosylation OR fucose AND oligosaccharides OR glycoproteins	lactation OR milk maturation	microbiota OR microbiome protection OR pathogen adhesion
breastfeeding AND fucosylation OR fucose AND oligosaccharides OR glycoproteins	gestational age OR week of delivery	infant formula OR donor milk

**Table 2:** Characteristics of milk oligosaccharides of some mammals.

	Human Milk HMOs	Bovine Milk BMOs	Goat Milk GMOs	Porcine Milk PMOs
Concentration	~20–25 g/L in colostrum and ~5–20 g/L in mature milk [15,16,17]	1–2 g/L in colostrum and ~0.05–0.1 g/L in mature milk [174,177]	1.1–1.3 g/L [176]	23 g/L in colostrum and 5–10 g/L in mature milk [178]
Number of identified structures	>200 [5,21,22,23]	over 50 [153]	29 [176]	33 [173]
Fucosylated structure (% of all structures)	35–50% [3]	<1% [153]	ok. 20% [176]	0.89–8.95% [174,178]
Concentration of 2′-FL	2.7 (1.88–4.9) g/L [3]	Absent [179] or present in low amounts [180]	1.12 mg/L [175]	present [173]

## Abbreviations

2′-FL	2′-fucosyllactose
3-FL	3-fucosyllactose
AGP	α1- acid glycoprotein
DC	dendritic cells
DC-SIGN	dendritic cell-specific intercellular adhesion molecule-3-grabbing non-integrin
DFL	difucosyllactose
DFpLNnH	difucosyl-para-lacto-N-neohexaose -
DSLNT	disialyllacto-N-tetraose
FN	fibronectin
Fuc	fucose
Gal	galactose
Glc	glucose
GlcNAc	N-acetylglucosamine
GOS	galacto-oligosaccharide
HMGs	human milk glycoproteins
HMOs	human milk oligosaccharides
LDFT	lactodifucotetraose
LF	lactoferrin
LNDFH I	lacto-N-difucohexaose I
LNFP I	lacto-N-fucopentaose I
LNFP II	lacto-N-fucopentaose II
LNFP III	lacto-N-fucopentaose III
LNnT	lacto-N-neotetraose
LNT	lacto-N-tetraose
Neu5Ac	sialic acid
S-IgA	secretory immunoglobulin A
